# Microbiological Aspects of Root Canal Infections and Disinfection Strategies: An Update Review on the Current Knowledge and Challenges

**DOI:** 10.3389/froh.2021.672887

**Published:** 2021-06-25

**Authors:** Jasmine Wong, Daniel Manoil, Peggy Näsman, Georgios N. Belibasakis, Prasanna Neelakantan

**Affiliations:** ^1^Discipline of Endodontology, Division of Restorative Dental Sciences, Faculty of Dentistry, The University of Hong Kong, Hong Kong, China; ^2^Division of Oral Diseases, Department of Dental Medicine, Karolinska Institute, Huddinge, Sweden

**Keywords:** biofilm, disinfection, irrigation, microbiome, metaproteome, metatranscriptome

## Abstract

The oral cavity is the habitat of several hundreds of microbial taxa that have evolved to coexist in multispecies communities in this unique ecosystem. By contrast, the internal tissue of the tooth, i.e., the dental pulp, is a physiologically sterile connective tissue in which any microbial invasion is a pathological sign. It results in inflammation of the pulp tissue and eventually to pulp death and spread of inflammation/infection to the periradicular tissues. Over the past few decades, substantial emphasis has been placed on understanding the pathobiology of root canal infections, including the microbial composition, biofilm biology and host responses to infections. To develop clinically effective treatment regimens as well as preventive therapies, such extensive understanding is necessary. Rather surprisingly, despite the definitive realization that root canal infections are biofilm mediated, clinical strategies have been focused more on preparing canals to radiographically impeccable levels, while much is left desired on the debridement of these complex root canal systems. Hence, solely focusing on “canal shaping” largely misses the point of endodontic treatment as the current understanding of the microbial aetiopathogenesis of apical periodontitis calls for the emphasis to be placed on “canal cleaning” and chemo-mechanical disinfection. In this review, we dissect in great detail, the current knowledge on the root canal microbiome, both in terms of its composition and functional characteristics. We also describe the challenges in root canal disinfection and the novel strategies that attempt to address this challenge. Finally, we provide some critical pointers for areas of future research, which will serve as an important area for consideration in Frontiers in Oral Health.

## Introduction

Root canal infections are caused by microorganisms that have penetrated the dental pulp and colonized the root canal system. As these microbial communities and their metabolic by-products rapidly gain access to periradicular tissues *via* apical or lateral foramina, they trigger a series of inflammatory responses. These responses cause the lysis of soft and hard periradicular tissues mostly as a result of the proteolytic activity of immune cells (neutrophils, macrophages, and mast cells) and the recruitment of osteoclasts [1]. This ultimately creates a bony cavity filled with cellular debris, cholesterol crystals, osteoclasts, fibroblasts, and various proportions of immune cells according to the level of inflammation [2]. Dead bacteria, and in some instances also live bacteria, are also found [3]. Such periradicular lesion triggered by the microbial communities within the root canal system is termed apical periodontitis. Apical periodontitis represents the main cause of dental emergency interventions [4, 5], and its exacerbated forms may spread to nearby facial spaces further leading to severe, life-threatening complications [6]. Reportedly, endodontic infections are responsible for nearly 7,000 hospitalizations per year in the US only [7]. On top of these adverse infectious evolutions, there is evidence supporting associations between chronic endodontic infections and systemic conditions such as diabetes mellitus or cardiovascular diseases [8–10].

Despite substantial progresses of modern endodontics with regards to mechanical instrumentation of radicular spaces, root canal infections, and their associated apical periodontitis lesions remain remarkably prevalent [11]. In fact, a recent systematic review has even reported an increase in the prevalence of apical periodontitis lesions during the last 8–9 years, reportedly due to inadequate endodontic and restorative treatments [12]. In Europe, the prevalence of apical periodontitis reaches 34–61% of individuals and affects between 2.8 and 4.2% of teeth, figures that increase in aging populations [13, 14]. Whereas, such infectious apical lesions can heal following root canal treatment, procedures that have not satisfactorily eliminated the microbial communities colonizing the root canal system are deemed to perpetuate persistent apical lesions [15–17]. Among Western populations, between 41 and 59% of individuals are estimated to have undergone at least one endodontic treatment, yet 24–65% of these treated root canals remain associated with radiographic evidence of persistent apical periodontitis [13, 14].

The ultimate goal of root canal treatment is to prevent the development of apical periodontitis, by removing infected and/or inflamed pulpal tissues and by creating the aseptic intraradicular conditions compatible with periradicular healing, if a lesion already exists. In essence, root canal treatment aims at eradicating the infection and further preventing microorganisms from re-penetrating the root canal system. In this perspective, a thorough understanding of the microbial-driven etiology of apical periodontitis, along with the antimicrobial challenges faced within root canals, is the foundation of the endodontic practice and provides the outline for effective therapy.

In this review, we focus on the latest microbial identification methods, based on high-throughput DNA sequencing to characterize the intra-radicular microbiota, and current antimicrobial approaches to tackle intra-radicular biofilms. We finally outline areas where further research may be warranted to improve our current understanding of the microbial ecology within root canals and its impact on oral and systemic health.

## Microbial Etiopathogenesis of Apical Periodontitis

Decades of research have established that the presence of bacteria in the root canal system is elemental for the development of apical periodontitis [18–20]. Although bacteria play an indispensable role in the etiopathogenesis, the pulp is generally guarded by layers of mineralized tissues, which help protect and maintain pulpal health and integrity. Bacterial invasion from the oral environment is only possible when there is a breach of the surrounding dentine, enamel and/or cementum, permitting entry of bacteria and its by-products, contaminating the originally sterile pulp. In fact, there are many potential pathways that may allow bacteria to invade the root canal system, the most common of which is dental caries. Other common routes of infection include cracks, trauma, exposed dentinal tubules, and iatrogenic causes [21].

As the root canal infection progresses, the pulpal status evolves from a healthy to a diseased state, eventually marked by necrosis. Although a vital pulp presents with a plethora of defense mechanisms to prevent its demise, an injured or necrotic pulp is much more vulnerable to bacterial invasion as the damaged vascular innervation and reduced number of viable cells strip it from its ability to carry out reparative and protective reactions [22, 23]. Given the low compliance nature of the pulpal environment and limited collateral circulation, the host's local defense systems are readily overwhelmed by the pathogenic challenge and this ultimately leads to necrosis [24]. The bacteria present in saliva, carious biofilms, and dental plaque morph into opportunistic microbes as they rapidly occupy the evolving ecological niche of the root canal system, forming multi-species communities. Their pathogenicity is driven by virulence factors such as lipopolysaccharides, proteases, exotoxin, and other secreted or structural components, with the former playing dominant role in the development of apical periodontitis [25]. Bacteria and their by-products then infiltrate the periradicular tissues through apical and lateral foramina, instigating inflammation and mineralized tissue resorption [26].

Root canal diseases are instigated by polymicrobial infections. The microbial composition of these bacterial communities evolves over time, fueled by the evolving root canal ecology and inter-species interactions. During the initial phases of the infection, the pulp presents with higher oxygen tension and an abundance of nutrients from oral cavity, allowing facultative bacteria to thrive. This also leads to greater bacterial counts and overall diversity [27]. On the other hand, the more carbohydrate depleted yet protein rich, anaerobic environment that present in more established infections, particularly in apical depths of the root canal, favors asaccharolytic, obligate anaerobes [28, 29]. Microbial succession is a dynamic process that can also be affected by iatrogenic alterations to the pulpal ecology, such as introduction of root canal disinfectants and changes to oxygen tension during the commencement of therapy. This may add to the selective pressure of specific species that thrive in harsh environments and have the potential to form persistent infections [30]. Several possible mechanisms that enable bacteria to persist include their ability to form biofilm, penetrate and remain in dentinal tubules, survive in nutrient depleted environments, possess virulence factors that can alter the host response and exhibit resistance to antimicrobial agents [29].

### Apical Periodontitis as a Biofilm-Mediated Disease

Our current understanding points toward the cumulative pathogenicity of a multi-species microbial community acting as a unit and the main driver in the pathogenesis of apical periodontitis [31]. These mixed infections primarily present in the root canal in the form of biofilms, which are not only found adhering to the intraradicular dentinal walls, ramifications and isthmuses, but also occasionally present on extraradicular root surfaces [32]. Ricucci and Siqueira [32] reported that biofilms were found in the overwhelming majority of canals associated with apical periodontitis, particularly those with large apical lesions. They can tolerate chemo-mechanical disinfection strategies and hide in anatomical complexities, contributing to recalcitrant root canal infections [33]. The microbial diversity contributes to their resilience against antimicrobial agents and is associated with persistent apical periodontitis [34, 35]. Furthermore, not only do the bacteria themselves contribute to the destructive processes of root canal diseases, but the components of the biofilm matrix can also encourage inflammation [36]. The root canal environment may similarly impact both the biofilm extracellular matrix and cellular composition. This was highlighted in a recent study which reported that biofilm age and exposure to collagen, saliva, and serum, differentially influenced the exopolysaccharide and protein content, as well as the number of colony forming units in monospecies *E. faecalis* biofilm models [37].

Biofilms are sessile communities of bacterial aggregates suspended in an extra-cellular fluid phase. These intraradicular biofilms are characterized by microbial microcolonies embedded in a self-produced matrix of extracellular polysaccharides, phosphoproteins, and eDNA infiltrated by fluid channels that allow circulation of nutrients and metabolites [38]. The matrix of extracellular polymeric substance (EPS) plays an important role in the form and function of biofilm, such as providing structural stability, enabling intercellular interactions, acting as a nutrient reservoir and offering protection against external stressors [39]. Furthermore, a key difference between biofilm bacteria and their planktonic counterparts is that they possess an altered phenotype which allows them to exhibit different characteristics such as enhanced pathogenicity and survival capabilities [40, 41].

In general, biofilm interactions can result in relationships of various dynamics that exert an impact on species selection. Positive relationships such as mutualism and commensalism promote the growth of certain species by way of synergistic nutritional webs, environment modification, genetic exchange, and so on. In contrast, negative interactions including competition for resources and amensalism deter the survival of others [42]. Furthermore, the close proximity and cell density of multiple microbial species enables for complex interactions that can alter the gene expression of the biofilm bacteria, which affects the biofilm characteristics and how it functions as a unit. Horizontal gene transfer may improve the overall biofilm survival by the inter-species exchange of genetic information responsible for virulence properties, adaptation to environment and resistance to environmental stresses and host defenses [43]. Quorum-sensing is another method of inter-microbial communication and involves the production and excretion of autoinducers, followed by activation of receptors which trigger or suppress specific genes that regulate virulence factors and biofilm formation amongst other things [41]. Overall, this results in biofilm-specific behaviors, which may not only enhance their pathogenicity but also heighten their antimicrobial resistance.

Biofilms are known to exhibit a superior tolerance to antimicrobial agents whilst compared to the more vulnerable planktonic equivalents, with some studies reporting a 1,000-fold difference [44, 45]. Apart from transfer of genetic material and cell-cell signaling, there are other advantages of the biofilm lifestyle that enhance the resistance against antimicrobial agents. Bacteria in biofilms benefit from the added protection from the EPS matrix, which shields them from the infiltration of antimicrobial agents [38]. Also, components of the biofilm matrix also can chemically react with antimicrobial agents to neutralize them [44]. Furthermore, biofilm bacteria exhibit reduced growth rates which prevent rapid uptake of antimicrobial agents [46]. Environmental stressors, such as nutrient scarcity and the exposure to antimicrobials, may induce gene expression for more resistant phenotypes and elicit a state of dormancy to give way to repopulation of antimicrobial-tolerant persister cells that can survive root canal treatment [47, 48].

### Microbial Profiles of Root Canal Infections

The term *microbiota* defines a collection of microorganisms that thrive within a defined ecosystem. The oral ecosystem harbors one of the richest microbiota of the human body, by far dominated by the domain Bacteria; although Archaea, fungi, viruses and Protozoa may also be found [49, 50]. To date, no <775 species-level bacterial taxa have been cataloged on the human oral microbiome database (HOMD) [51]. Because root canal infections most often occur as a sequel to caries or trauma, the bacterial communities that initially colonize the pulpal space result from an ecological selection of the oral microbiota [52, 53]. The composition of the endodontic microbiota therefore derives from a compositional shift of oral communities, driven by the specific ecological conditions of infected root canals.

Endodontic infections can be didactically subdivided into three categories evocative of the time when microorganisms entered the pulpal space [54]. Primary endodontic infections are caused by microorganisms involved in initial pulp invasion and subsequent colonization of necrotic tissues. Secondary endodontic infections are caused by microorganisms introduced into the root canal secondarily to professional intervention, either iatrogenically during operative procedures, or later by coronal percolation *via* micro-leaking restorations. Persistent endodontic infections are caused by microorganisms that are part of either a primary or secondary infection, that resisted chemo-mechanical debridement procedures and survived within the scarce environment of treated root canals. Of note, because persistent and secondary infections remain clinically challenging to distinguish, they tend to be regrouped under the same pathological entity [55].

#### Targeted Microbial Identification in Root Canal Infections

For many years, knowledge of the root canal microbiota originated mostly from culture techniques and closed-ended molecular approaches such as fluorescent *in situ* hybridization (FISH), DNA-DNA hybridization checkerboards or PCR and its variations [56–59]. These approaches permitted to identify sets of bacterial taxa that appeared distinct between root canal infection types. Primary infections appeared dominated by 40–50 Gram-negative strictly anaerobic species of the genera *Fusobacterium, Prevotella, Porphyromonas, Tannerella, Treponema* [55, 60, 61]. Persistent/secondary infections seemed to harbor less diverse microbial communities composed of 10–20 taxa, mostly Gram-positive facultative anaerobes including species of *Streptococcus, Lactobacillus, Actinomyces*, and *Enterococcus* [17, 62]. *Enterococcus faecalis* particularly was frequently retrieved from persistent/secondary infections and yet remained rarely detected in primary infections [63–65]. These observations demonstrated the ability of *E. faecalis* to cope with the bleak conditions of treated root canals, and advocated for an important pathogenic role in secondary infections [66].

Together, conventional approaches provided a substantial body of information on the microbial composition and diversity of the different endodontic infections, albeit undermined by inherent limitations. Whereas, culture techniques fail to identify the as-yet-uncultivated microbiota, the pre-selection of primers and probes typical of closed-ended molecular methods skews microbial identification toward pre-targeted species and precludes the detection of less studied or “unexpected” taxa.

#### Next-Generation Profiling of the Intraradicular Microbiome

During the last decade, next-generation sequencing (NGS) technologies, that allow the high-throughput sequencing of DNA, have found unquestionable relevance to study complex microbial communities [67, 68]. In endodontics, microbial identification has mostly relied on the partial sequencing of the 16S ribosomal RNA (16S rRNA) gene [69]. This gene bares the advantages of being ubiquitous among bacteria and to combine slowly-evolving (conserved) regions, that can be employed to detect virtually all taxa in a sample, along with fast-evolving (variable) regions, that differ between taxa and become valuable targets for bacterial identification [70].

Typically, 16S amplicons that span one or several variable regions are sequenced thousands of times in parallel (5,000–50,000 coverage depth), and then employed to infer bacterial taxonomy by comparison with reference databases. NGS profiling of root canal communities unveiled a previously-hidden microbial diversity, substantially expanding the catalog of bacteria identified above 400 taxa on average [69]. Furthermore, such important diversity was similarly observed in both primary and persistent/secondary infections, which is contrasting with classical reports [71–75]. As a result of this important diversity, bacterial taxa were no longer unequivocally specific to an infection type, rather, the same taxa tended to be identified in all root canal infections, albeit in different abundances. The root canal microbiota was therefore deemed to be compositionally unspecific, yet differentially abundant in each infection type. Table 1 provides an overview of the main characteristics and findings of the NGS-based studies included herein.

**Table 1:** Overview of the studies included that investigated the root canal microbiota by means of 16S rRNA gene next-generation sequencing.

**Study**	**Santos et al. [76]**	**Siqueira et al. [77]**	**Hsiao et al. [52]**	**Özok et al. [78]**	**Hong et al. [71]**
**I**					
Microbial outcome	Asymptomatic PIs (roots) vs. symptomatic AA (pus)	PIs' microbiota	Oral swabs vs. AA-associated roots and pus	Coronal vs. apical root segments in PI's	PIs vs. SPI
16S variable regions	V4	V1–V3	V1–V2	V5–V6	V1–V3
Taxa id. (OTUs)	916	187	325	606	803
Sampling method.	*In vivo*: paper points and abscess aspiration	*Ex vivo*: cryo-pulverized roots	*In vivo*: paper points and abscess aspiration	*Ex vivo:* cryo-pulverized roots	*In vivo*: paper points
Main findings	13 phyla subdivided into 67 genera. 20 genera exclusively in pus from AAs and 18 in PIs. 18% of the 165 OTUs detected in both pus and PI-associated root canal.	84 genera belonging to 10 phyla detected. Majority were low-abundant OTUs. Important interindividual variation in the composition of the apical microbiota.	11 Phyla detected. Lower bacterial diversity in AAs root canals and pus than in oral swabs. *Streptococcus* spp. most abundant in oral swabs, *Prevotella* and *Fusobacterium* most abundant in AAs root canals and pus.	24 bacterial phyla detected, Proteobacteria more abundant in apical segments and Actinobacteria more abundant coronal. Apical segments displayed higher diversity than the coronal segments.	10 phyla subdivide into 148 genera. No significant differences in bacterial composition between PIs and SPI.
Main phyla (max. top 6)	In PIs: Firmicutes (59%), Bacteroidetes (14%), Actinobacteria (10%), Fusobacteria, Proteobacteria, Spirochetes.	Proteobacteria (43%), Firmicutes (25%), Fusobacteria (16%), Bacteriodetes (9%), Actinobacteria (5%), Synergistetes.	Firmicutes (21.6%), Bacteroidetes, Fusobacteria, Proteobacteria, Actinobacteria, Synergistetes. TM7, Tenericutes, Deinococcus-Thermus, SR1, Euryarchaeota, and Cyanobacteria.	Firmicutes (48%), Actinobacteria (30%), Bacteroidetes (12%), Acidobacteria, BRC1, Chlamydiae.	In both infection types: Bacteriodetes (29.6%), Firmicutes (23.2%), Actinobacteria (10.5%), Fusobacteria (13.1%), Protobacteria (8.8%), Synergistetes (6.3%).
Main genera/species (max. top 12)	In PIs: *Phocaeicola* (12.5%), *Eubacterium* (12%) and *Pseudoramibacter* (10%). Most abundant genera from pus sampling: *Fusobacterium* (19%), *Parvimonas* (11%) and *Peptostreptococcus* (10%).	*Fusobacterium* (15%), *Pseudoramibacter* (8%), *Novosphingobium* (8%), *Ralstonia* (6%), *Bacterioides* (5%).	*Fusobacterium, Prevotella, Granulicatella, Eubacterium, Streptococcus, Porphyromonas, Afipia, Phocaeicola, Veillonela, Parvirmonas, Gemella, Pyramidobacter*.	*Lactobacillus* (14.3%), *Actinomyces* (11.9%), *Streptococcus* (0.4%), unclass. *Actinobacteria* (6.9), *Prevotella* (6.1%), *Parvimonas* (3.4%), *Pseudoramibacter* (3%), Order Bacteroidales (2.7%), Fam. Veillonellaceae (2.5%), *Fusobacterium* (2%), *Peptostreptococcus* (2%), *Porphyromonas* (1.8%).	In PIs: *Prevotella, Propionibacterium* and *Pyramidobacter*. In SPI: *Fusobacterium, Porphyromonas* and *Prevotella*.
**II**					
Microbial outcome measured	Symptomatic vs. asymptomatic SPIs	PIs vs. SPIs vs. AAs	PIs vs. SPIs.	PIs vs. periodontal pockets in cases of endo-perio lesions	Apical segments of SPIs
16S variable regions	V1–V2	V6	V1–V2	V3–V4	V4
Total taxa id. (OTUs)	741	PIs: 36; SPIs: 43; AAs: 45	339	289	538
Sampling method.	*In vivo:* paper points	*In vivo:* paper points	*In vivo:* paper points	*In vivo:* paper points	*Ex vivo:* cryo-pulverized roots
Main findings	Symptomatic SPIs had more Firmicutes and Fusobacteria than asymptomatic ones. In turn, asymptomatic SPIs exhibited more Proteobacteria and Actinobacteria.	Highly diverse microbiota in all infection types. *E. faecalis* identified only in SPIs. One AA sample displayed a significantly high proportion (47%) of Proteobacteria (*Janthinobacterium lividum)*.	Bacteroidetes most abundant phylum in both infection types. Different bacterial compositions in symptomatic and asymptomatic infections. SPIs significantly enriched with Proteobacteria and Tenericutes as compared to PIs. 14 genera differentially abundant between PIs and SPIs.	Firmicutes most abundant in all sampling sites. *Desulfobulbus* sp. oral taxon 041 was associated w/periapical lesions ≤ 2 mm.	11 phyla subdivided into 103 genera. *Fusobacterium* and *Pseudomonas* dominated. *Enterococcus* spp. found in 4 cases, relatively low-abundant.
Main phyla (max. top 6)	Firmicutes (29.9%), Proteobacteria (26.1%), Actinobacteria (22.72%), Bacteroidetes (13.31%) and Fusobacteria (4.55%).	In all infection types: Firmicutes, Actinobacteria, Bacteriodetes, Fusobacteria, Proteobacteria, Synergistetes.	In all infection types: Bacteroidetes (36.2%), Firmicutes (32.9%), Actinobacteria (8.1%), Synergistetes (7.4%), Fusobacteria (7.4%), Proteobacteria (5.2%).	In PIs: Firmicutes (75.09%), Proteobacteria (7.85%), Actinobacteria (7.01%), Bacteroidetes (6.77%)	Proteobacteria (46%), Firmicutes (18%), Fusobacteria (15%) and Actinobacteria (8%)
Main genera/species (max. top 12)	*Streptococcus* (10.9%), *Prevotella* (8.21%), *Lactobacillus* (8.06%), *Kocuria* (5.17%), *Neisseria* (3.38%), *Enterococcus* (2.59%), *Acinetobacter, Atopobium, Rothia, Pseudomonas, Propionibacterium, Schlegelella*.	No relative abundances were reported for any of the genera detected.	PIs enriched with: Bacteroidaceae_unclassified, *Pyramidobacter, Parvimonas*. SPIs enriched with: *Fusobacterium*, Bacteroidaceae_unclassified, and *Prevotella*.	In PIs: *Enterococcus faecalis, Parvimonas micra, Bacteroidaceae* [G-1] sp. oral taxon 272, *Peptostreptococcaceae* [G-1] sp. oral taxon 113, *Mogibacterium timidum, Peptostreptococcus stomatis, Filifactor alocis*, and *Fretibacterium fastidiosum*.	*Fusobacterium* and *Pseudomonas* (15%), *Klebsiella, Stenotrophomonas, Pseudoramibacter, Pyramidobacter, Enterococcus*.
**III**					
Microbial outcome	In PIs: microbiota vs. mycobiome and coronal vs. apical root segments	PIs vs. SPIs	SPIs	PIs vs. aSPIs	
16S variable regions	V3–V4	V4	V3–V5	V3–V4	
Total taxa id. (OTUs)	338	Not specified	152	347	
Sampling method.	*Ex vivo:* cryo-pulverized roots	*Ex vivo:* cryo-pulverized roots	*In vivo:* paper points	*Ex vivo:* intraradicular content collected with endodontic files	
Main findings	Coronal and apical root segments had similar microbiome and mycobiome. No correlations between microbiota and mycobiomes.	160 genera belonging to 15 phyla were detected. PIs and SPIs displayed no significant differences in microbiota composition.	125 bacterial species belonging to 68 genera and 9 phyla were detected.	18 phyla subdivided into 177 genera. Microbiotas were differentially abundant in each infection type. Co-occurrence analysis demonstrated microbial interactions specific to each infection type.	
Main phyla (max. top 6)	No phylum level assessment	Proteobacteria (33.4%), Firmicutes (32.3%), Bacteroidetes (26.3%), Fusobacteria (4.2%), and Actinobacteria (2.9%)	Prior to disinfection: Firmicutes (47%), Fusobacteria (14%), Bacteroidetes (12%), Proteobacteria (12%), Actinobacteria (9%), Synergistetes (4%)	Firmicute (PI 36/SPI 48.4%), Bacteroidetes (PI 23.8/SPI 9.5%), Actinobacteria (PI 6.4/SPI 23.4%), Fusobacteria (PI 16/SPI 5.6%), Synergistetes (PI 9.9/SPI 4.5%), Proteobacteria (PI 2.4/SPI 4.8%)	
Main genera/species (max. top 12)	PIs-microbiota: *Prevotella* (12.7%), *Lactobacillus* (11.2%), *Actinomyces* (7.5%), *Fusobacterium* (7.2%), *Atopobium* (6.9%), *Streptococcus* (4.4%), *Leptotrichia* (4.3%), *Phocaeicola* (3.5%), *Pyramidobacter* (2.9%), *Porphyromonas* (2.7%)	*Prevotella* (PI 23.5%/SPI 15.7%), *Porphyromonas* (16.5% mean PI-SPI), *Neisseria* (13.2% mean PI-SPI), *Lactobacillus* (11.7% mean PI-SPI), *Parvimonas* (11.1% mean PI-SPI), *Streptococcus* (PI 9.4%/SPI 12%), *Enterococcus* (PI 2%/SPI 5%), *Camplyobacter* (PI 2%/SPI 0.1%), and *Granulicatella* (1% mean PI-SPI)	Prior to disinfection: *Enterococcus* (13.9%), *Fusobacterium* (12.7%), *Streptococcus* (9.8%), *Actinomyces* (8.2%), *Desulfobulbus* (5.2%), *Fretibacterium* (3.6%), *Treponema* (2.3%), *Prevotella* (2%), *Alloprevotella* (0.01%)	*Fusobacterium nucleatum* (PI 16/SPI 5.3%), *Enterococcus faecalis* (PI 0.01/SPI 18.9%), *Parvimonas micra* (PI 8/SPI 2.6%), *Porphyromonas endodontalis* (PI 5.7/SPI 2%), *Streptococcus constellatus* (PI 0.6/SPI 3.5%), *Slackia exigua* (PI 0.7/SPI 1.3%), *Schwatzia* AF287291 (PI 1/SPI 3.5%), *Dialister pneumosintes* (PI 3.4/SPI 1.2%), *Prevotella oris* (PI 5.7/SPI 1.5%)	

*AA, apical abscess; OTU, operational taxonomic unit; PI, primary infection; SPI, secondary/persistent infection*.

Overall, Firmicutes, Bacteroidetes, Proteobacteria, Actinobacteria, and Fusobacteria were the most abundant phyla detected in root canal infections, regardless of the type [69]. At lower taxonomic levels, primary infections were frequently confirmed to harbor higher abundances (>5%) of previously identified genera such as *Fusobacterium, Pseudoramibacter, Porphyromonas, Prevotella, Lactobacillus, Bacteroides*, or *Ralstonia* [77, 78, 80, 82]. These taxa were frequently detected along with strictly anaerobic, proteolytic Firmicutes such as *Pseudoramibacter alactolyticus, Parvimonas micra* and asaccharolytic species of *Dialister*, such as *D. pneumosintes* or *D. invisus*. NGS studies also disclosed the involvement of previously little-known taxa, such as the Clostridales species *Mogibacterium timidum*, seemingly enriched in primary infections [75, 80].

In turn, persistent/secondary infections appeared enriched with Synergistetes representatives including *Pyramidobacter piscolens* and *Fretibacterium fastidiosum* [75, 81, 83]. Other genera detected in increased abundances in persistent/secondary infections included *Streptococcus, Prevotella, Lactobacillus, Desulfobulbus, Kocuria, Neisseria*, and *Enterococcus* [75, 79, 83]. The role of *E. faecalis* in persistent/secondary infections has been challenged by NGS reports. Unlike classical studies that reported prevalence values of *E. faecalis* in cases of treated root canals reaching up to 90%, the species was detected in significantly lower frequencies (~30%) by 16S-sequencing in persistent/secondary infection [72, 74, 75, 81, 83]. Nonetheless, when present, the species could reach intriguingly high abundance ranging from 14 up to 90% [75, 81, 83]. Because evidence shows that *E. faecalis* is more frequently retrieved from root canals after multiple visits or temporarily left open, it has been purported that the species is a secondary opportunistic colonizer in treated root canals rather than a persister from unsuccessfully treated primary infections [84, 85]. In such case, one could speculate that treated root canals displaying remarkably high abundance of *E. faecalis* may reflect secondary infections as opposed to persistent ones, that would in turn be associated with a higher microbial diversity. In this line, it has been shown using *in vitro* biofilm models that when opportunistically present among other common oral species, it can grow in a predominant manner within the biofilm [86].

Few studies have also attempted to discriminate the microbiota of symptomatic and asymptomatic presentations, both in cases of primary or persistent/secondary infections. Overall, the microbiota of symptomatic primary root canal infections tended to be more diverse than asymptomatic ones [73, 76]. This trend appears reversed in persistent/secondary infections, in which decreased diversity was associated with asymptomatic clinical presentations [73, 79]. These results are nonetheless to be considered with caution, as the comparison of samples collected from root canals or obtained by pus aspiration may raise some methodological concerns [76].

Taken together, NGS-based community profiling has confirmed the involvement of previously identified species, and further expanded the list to a myriad of low abundant taxa with yet-poorly understood clinical relevance. From an ecological standpoint, however, every taxon in a mixed consortium is important to detect regardless of its abundance, as any taxa may act as keystone species and potentiate the pathogenicity of the entire community [53, 87]. In this perspective, the relatively short length of the 16S amplicons used by current 16S rRNA-sequencing approaches (roughly 400 bp) is an important limitation, as it limits taxonomic profiling to the genus level [88]. Although advances in long read technologies are expected to soon allow the full length-sequencing of the 16S rRNA gene and improve taxonomic resolution [89, 90], significant taxonomic and functional characteristics would remain concealed at the strain-level [91]. One element of answer may stem from shotgun metagenomics, that can re-assemble complete metagenomes to identify strain-level changes and predict the functional genetic potential of microbial communities [92]. Besides, to better grasp the metabolic processes and functional networks that underlie pathogenicity, assessment of the microbial genes that are actively transcribed (metatranscriptomics) and/or translated (metaproteomics) is also necessary [93, 94].

### Insights Into the Metaproteome of Root Canal Communities

Because proteins are function effectors, proteomic approaches that examine the collection and abundance of proteins within microbial communities, i.e., the metaproteome, inform on the ongoing biological processes. Thus far, only few studies have relied on metaproteomics to functionally explore the processes engaged in bacterial cells during endodontic infections [95–98]. Although these studies remain preliminary due to the limited number of samples (between 7 and 20 per study), and the considerable variations in sampling methods (pus aspiration from abscesses, intra-radicular sampling with paper points, apical lesions, and root fragments resected during surgery), their findings provided invaluable and novel insights into the mechanisms of bacterial survival and virulence within root canals. Intriguingly, housekeeping processes were among the most abundant functional categories regardless of the sample type [96–98]. This indicates the presence of metabolically active bacteria in both necrotized and treated root canals, and further supports the concept of active bacteria able to invade periradicular tissues, as evidenced by analyses of surgically retrieved apical lesions [97].

Aside from housekeeping functions, several other proteins were related to pathogenic processes. Abundantly detected enzymes and adhesins, such as fibrinogen-binding protein, fibronectin-binding A domain, polysaccharide lyases or glycosyltransferase I (exopolysaccharide producing enzyme), are testimony of bacterial adhesion and biofilm formation [99–101]. Equally crucial to pathogenicity, are the numerous proteolytic enzymes detected including collagenases, metalloproteases, serine proteases, and extracellular peptidases [95–98].

During the course of an infection, proteolytic activities serve to break-down peptides and acquire amino acids, to degrade connective tissues for host invasion or to cleave antibodies and complement molecules for immune evasion [102–104]. Also, a series of antibiotic-resistance factors have also been recurrently detected in endodontic communities. These typically included TetRA, conferring resistance to tetracycline, β-lactam-degrading enzymes (β-lactamase), multidrug efflux pumps (MdtB), and multiple transcriptional regulators of antibiotic-resistance genes (AsnC-type, LacL-type, and QseB-type) [95–98]. Because systemic antibiotics become indispensable when endodontic infections spread into topical tissues or systemically, these findings are clinically troublesome [105, 106]. Finally, numerous stress response proteins including the chaperonins GroEL, DnaK, or HslU along with the nucleotide excision repair complex UvrABC were common findings [95–98]. This is unsurprising as expression of stress response proteins is often co-regulated with virulence factors and antibiotic resistance genes.

Importantly though, in addition to increasing tolerance limits, stress response proteins may also exhibit cytotoxicity and potentiate pathogenicity by promoting the secretion of pro-inflammatory cytokines [107, 108]. The proinflammatory response in the pulpal and periradicular region is an important histopathological hallmark of endodontic infections, that may take far beyond this review to discuss. Worth highlighting here, is the significance of the receptor activator of NF-κB ligand-osteoprotegerin system in the periradicular osteolysis that may accompany endodontic infections [109], which may in turn be differentially regulated depending on the endodontic diagnosis [110].

## Clinical Implications of Root Canal Infections

### Challenges in Eliminating Root Canal Biofilms

While the understanding of the microbial complexity of root canal infections has substantially improved over the past decade. On the other hand, although the success rates of root canal treatments have been very favorable, they have not experienced the same extent of improvement, specifically in teeth which had a necrotic pulp with or without a periradicular lesion pre-operatively [111, 112]. From a microbiological standpoint, this is unsurprising—infected teeth have densely colonizing biofilms that are strongly adherent to the dentin, posing challenges in removal. However, an additional, yet exemplary challenge appears in the form of anatomical complexities in the root canal system—inter and intra-canal communications/isthmi, lateral and accessory canals, apical delta—are a norm rather than an exception. Therefore, there are two key challenges in removing root canal biofilms: microbiological and anatomical.

As discussed earlier, biofilms are inherently tolerant to antimicrobial agents. Biofilms are surface-attached communities where the bacterial and/or fungal cells are held together in a highly cohesive matrix, which presents a diffusion limitation to antimicrobials. Therefore, for an antimicrobial agent to effectively kill the microbial cells, it should be able to dissolve and disrupt the matrix. Alternatively, this matrix should be disrupted by mechanical means to allow antimicrobial chemicals to reach the cells housed inside the biofilm matrix. While this appears simple in theory, the clinical practicalities are complicated by the rather “blind” area of the root canal system that we work in and the complex myriad of inter-communicating pathways which are never accessible to mechanical instrumentation.

Despite such knowledge of the so-called biofilm entity, it is notable that most of the reported studies on anti-biofilm agents, including traditional disinfectants such as sodium hypochlorite or antimicrobials such as chlorhexidine and calcium hydroxide, do not characterize the effects on biofilm composition. Such as the constituents of the self-produced EPS, which includes various extra-polysaccharides, eDNA, and phosphoproteins. The widely reported experiments in this regard, include counting the number of microbes after treatments using the plate count method or qPCR and characterizing the “architecture” of biofilms using three-dimensional imaging approaches such as confocal laser scanning microscopy. However, such studies on confocal microscopic examination routinely use fluorophores that stain the live and dead microbial cells but not the matrix. While one may assume that the disinfectants should have likely disrupted the matrix to reach the microbial cells, this should be demonstrated with experimental evidence. Furthermore, such an assumption may not be true. Tawakoli et al. [113] characterized the effects of 5% sodium hypochlorite, 17% EDTA, 3% hydrogen peroxide, and 2% chlorhexidine on a three-species biofilm and reported that sodium hypochlorite eradicated the stainable matrix and bacteria in cultured biofilms, while chlorhexidine and hydrogen peroxide merely reduced bacterial cell volumes without dissolving the matrix [113]. Ali et al. [114] demonstrated that a natural molecule trans-cinnamaldehyde could kill *E. faecalis* biofilm cells as effectively as sodium hypochlorite. Confocal microscopic characterization demonstrated largely “red” areas in the treated biofilm, implying complete bacterial killing [114]. However, when the same authors characterized the polysaccharide matrix component, they discovered that trans-cinnamaldehyde could not eliminate the matrix.

The above summary results in a call for action. The authors of this review strongly recommend that when traditional or novel disinfectants are tested on biofilms, both the cellular and extracellular content of the biofilm must be studied to provide comprehensive information on anti-biofilm effects. Such characterization of the extracellular content maybe performed using biochemical methods [114, 115] or by using matrix-component specific fluorophores for confocal microscopic imaging [37]. Furthermore, the use of multi-species as well as aged-biofilm models may better represent the biofilms found in infected root canals, which could enhance the applicability of *in vitro* research to the clinical situation.

From the context of anatomical challenges, while instrumentation of the root canal with stainless steel or nickel titanium hand or engine-driven instruments may help in removing biofilms from root canal walls, much is required to eliminate biofilms and reduce microbial loads from the anatomical eccentricities [33, 116]. The goal of activated irrigation is to mechanically flush the irrigating solutions such as sodium hypochlorite into such eccentricities in addition to chemically enhancing their ability to dissolve organic matter [117, 118]. In this regard, there has been wide confusion over the use of agitation and activation strategies. While this is certainly important, such clarification of terminology is not within the scope of this review. That said, several means of “dynamic” irrigation such as ultrasonic, sonic, multisonic, apical negative pressure, and laser have been interrogated to demonstrate their ability on eliminating biofilms and/or killing bacterial cells. Indeed, such adjunctive strategies are considered to improve irrigant infiltration into anatomical complexities [119].

One systematic review concluded that ultrasonic activation of irrigants with ultrasonic results in superior bacterial reduction from the root canal systems compared to other methods of irrigant activation and conventional syringe irrigation [120]. By contrast, another systematic review reported that there was insufficient evidence to conclude that ultrasonics resulted in superior bacterial reduction from root canals [121]. New multisonic approaches such as Gentlewave (Sonendo, Laguna Hills, CA, USA) have been shown to significantly reduce bacterial counts compared to ultrasonic [122]. That said, none of the studies that investigated the activation strategies reported the effects of activation on biofilm matrices or truly complex multi-species biofilms. Furthermore, the substantial heterogeneity in terms of irrigant concentration, duration, activation cycles preclude meaningful extrapolation to provide clinically relevant conclusions.

### Contemporary Root Canal Disinfection Strategies

#### Root Canal Irrigants

Irrigants are used to flush out debris, lubricate the canal and aid the elimination of root canal infection. Sodium hypochlorite is the most commonly used irrigant in endodontics due to its antimicrobial and tissue dissolving properties [123–125]. It has been suggested that 0.5 and 5% sodium hypochlorite were equally effective in reducing bacterial load when evaluated using culture studies [123]. However, other studies suggest the removal of *E. faecalis* in both biofilm and planktonic forms with sodium hypochlorite may be concentration dependent, with higher concentrations resulting in better outcomes [126, 127]. On the other hand, high concentrations of sodium hypochlorite, such as 5 or 9%, may result in the disintegration of the organic dentine matrix [128]. Such concentrations may be severely caustic to the apical tissues, particularly if extruded out the apical foramen [129]. Furthermore, needle irrigation with sodium hypochlorite may only be able to eliminate the bacterial residing in the more superficial regions of dentinal tubules [130]. Other than concentration, other factors can also affect its antimicrobial efficacy such as irrigant refreshment, volume, exposure time, flow, wall shear stress created and the method of delivery [131].

Bisbiguanide solutions are also commonplace in irrigation strategies, such as chlorhexidine digluconate, which is a frequently used disinfectant in endodontics. Chlorhexidine has been advocated as a final rinse irrigant due to its broad-spectrum antimicrobial activity, substantivity and its ability to inhibit collagen degradation [125, 132–134]. However, several drawbacks of its use include the incapacity to degrade organic tissue and a potential deleterious impact on periapical health outcomes, hence its use throughout endodontic treatment, particularly alongside sodium hypochlorite, is not recommended [135]. Furthermore, although chlorhexidine's ability to eradicate *E. faecalis* has been demonstrated in several studies [136], such effects appears to be limited to planktonic cells and its efficacy decreases in the presence of mature biofilm structures, permitting biofilm recovery [137, 138]. Although a recent meta-analysis of randomized controlled trials comparing sodium hypochlorite and chlorhexidine concluded that their antimicrobial efficacy was comparable, the authors highlighted the heterogeneity in bacterial detection methods within the included studies and also did not address the antibiofilm efficacy of the two irrigants [139]. It has been suggested the addition of 0.2% cetrimide, a cationic surfactant agent, may be able to act synergistically with 2% chlorhexidine to enhance antibacterial efficacy and improve substantivity [140]. Other emerging antiseptic solutions include alexidine and octenidine hydrochloride, which have been shown to exhibit antimicrobial effects similar to and greater than sodium hypochlorite, respectively [141].

Chelating agents are used in irrigation protocols to dissolve smear layer, remove mineralized debris and calcifications, and disrupt biofilm. Various acidic chelators exist, including such as ethylenediaminetetraacetic acid (EDTA), maleic acid, citric acid, and phosphoric acid [142, 143]. EDTA in particular has been recommended as an chelating irrigant due to its capacity to predictably remove the smear layer [125, 142, 144] and detach biofilm cells [145]. Pairing it with sodium hypochlorite has been shown to enhance the antibiofilm effects against *E. faecalis* biofilm [146]. A disadvantage is that excessive use may heavily demineralize dentine and weaken the root structure [147]. When coupled with sodium hypochlorite, it resulted in the loss of the mineral encapsulation and subsequent dissolution of the exposed collagen fibers [148]. The antibiofilm effects of EDTA and other chelators requires more investigation, using complex biofilm models.

Irrigants that combine chelating agents with antimicrobials have been developed in order to provide “one-stop” solutions for root canal disinfection. Several commercially available combination irrigants include BioPure® MTAD® (Dentsply Tulsa Dental Specialties, Tulsa, OK, USA), QMix® 2in1 (Dentsply Tulsa Dental Specialties), SmearOFF™ (Vista™ Dental Products, Racine, WI, USA). MTAD®, a mix of doxycycline, citric acid and a detergent, was developed as a final rinse irrigant [149]. It has been shown to possess antibacterial activity against *E. faecalis* [149, 150]. However, evidence suggests that it may not be superior than a final rinse of sodium hypochlorite and EDTA [151]. Qmix®, a formulation of chlorhexidine, EDTA and a detergent, was reported to be more effective than 2% chlorhexidine and MTAD® against planktonic and biofilm forms of *E. faecalis* [152]. Its antibacterial effectiveness against *E. faecalis* was similarly reported on by recent systematic review of *in vitro* studies [153]. The recently developed SmearOFF™ likewise contains chlorhexidine, EDTA and a surfactant, however an *in vitro* study suggested it had poor antibiofilm properties against *E. faecalis* [141].

Unlike the current final rinse protocols and solutions that utilize strong chelating agents such as EDTA, formulations combining antimicrobials with weak chelators offer the option of continuous chelation, which would be used throughout the cleaning and shaping process. EDTA is not only a strong cation chelator, but it also unfavorably reacts with sodium hypochlorite and reduces the chlorine content [154]. Therefore, a weak chelating agent, etidronic acid, also known as 1-hydroxyethylidene-1,1-bisphosphonate or HEBP, has been combined with sodium hypochlorite to form a solution for continuous chelation [155]. The suggested concentration ranges between 7 and 18% [154, 156]. When the combination of HEBP and sodium hypochlorite was applied to root dentine, it was able to remove the smear layer without excessive decalcification [156], although a recent study reported that it negatively affected the dentine nanohardness [157]. This solution also appears to possess commendable antibiofilm activity against *E. faecalis* biofilm [158] but this may be influenced by its short-term storage ability [159]. A clinical study evaluated a recently developed commercially available continuous chelator i.e., Dual Rinse® HEDP (Medcem GmbH, Weinfelden, Switzerland) and reported comparable antimicrobial activity to sodium hypochlorite used in isolation [160].

Recent studies have proposed clodronate, also a first-generation bisphosphonate, as another candidate for continuous chelation. Clodronate combined with sodium hypochlorite was found to effective in removing the smear layer [161]. It appeared to be better than solutions with etidronate at dissolving organic tissue [162]. However, evidence of its antimicrobial and antibiofilm efficacy has yet to be reported in the literature.

#### Intracanal Medicaments

The placement of an interappointment dressing has been advocated to provide a continuous supply of antimicrobial agents, restrict bacterial growth, and create a barrier for bacteria recolonization. Historically, a variety of intracanal medicaments have been suggested including calcium hydroxide, iodine potassium iodide, eugenol, formocresol, phenolic compounds, and various antibiotics [163]. Whilst several of these medicaments have fallen out of favor due to concerns of potential mutagenic and allergenic effects, calcium hydroxide remains as a first line intracanal medicament for necrotic cases with established infections [135, 164]. Multiple classic studies have shown its ability to reduce bacterial load due to its highly alkaline, tissue dissolving, and antimicrobial properties [124, 165, 166].

However, the efficacy of calcium hydroxide has also been questioned, particularly against bacteria implicated in recalcitrant root canal infections, such as *E. faecalis* and *Candida albicans* [167–170]. It also exhibits limited ability to adequately disinfect dentinal tubules [171] and has the potential to weaken the root structure if used over long periods of time [172]. There is conflicting and insufficient evidence on modifying calcium hydroxide with chlorohexidine to improve its antibacterial effects [173–175]. On the other hand, Zehnder et al. [176] reported that a combination of calcium hydroxide and sodium hypochlorite exhibited significantly better tissue dissolving properties and antimicrobial efficacy compared to mixtures using saline. The same study also found favorable properties with calcium hydroxide-chlorhexidine paste, although the effects were relatively short-lived [176].

A range of antibiotics and their combinations have been proposed as alternative intracanal medicaments since the mid-twentieth century [177]. More recently, triple antibiotic paste (TAP), consisting of metronidazole, ciprofloxacin, and minocycline, has been enthusiastically advocated for root canal disinfection prior to regenerative endodontic strategies [178, 179] due to its reportedly superior antibiofilm properties and ability to disinfect dentinal tubules compared to calcium hydroxide [180–182]. However, an *in vitro* study found TAP was only effective against *E. faecalis* biofilm and not against *Candida albicans* biofilm [170]. Furthermore, some guidelines challenge the evidence supporting its routine use, especially given the risks dentine discolouration, antibiotic resistance [164] and potential detriment to stem cells [183].

Antibiotics have also been combined with anti-inflammatory drugs in order to provide a dual therapeutic effect. Ledermix® paste (Lederle Laboratories, Seefeld, Germany), which consists of triamcinolone and a demeclocycline, has been proposed as an intracanal medicament for symptomatic teeth and concurrent endodontic and periodontal lesions due to the corticosteroid component [184]. However, Ledermix® on its own has limited antibacterial and antibiofilm effects against *E. faecalis* [169]. Some have suggested combining Ledermix® with calcium hydroxide to augment the antibacterial efficacy [169, 185], although this remains controversial with other studies refuting such benefits [186, 187].

### Novel and Advanced Disinfection Therapies

#### Nanoparticles

In the last decade, nanoparticle-based disinfection therapies have generated significant enthusiasm within the endodontic research community. Nanoparticles measure between 1 and 100 nm [188]. Their miniscule dimensions and large surface area to volume ratio mean they exhibit greater antimicrobial activity than their macroscale counterparts and are able to better infiltrate biofilm. They can also be functionalized with other compounds, such as photosensitizers, bioactive molecules, and drugs, to produce synergistic effects [189].

Silver nanoparticles are the most widely investigated metallic nanoparticle in the field of root canal disinfection strategies owing to their antibiofilm, antimicrobial, and antifungal properties [190]. A study found that a silver nanoparticle-based irrigant had antibiofilm activity comparable to 2% chlorhexidine and 5% sodium hypochlorite [191]. However, the antimicrobial efficacy of silver nanoparticles may be contact and time-dependent, hence their application as intracanal medicaments or root canal sealers may be more appropriate [189, 192]. There are concerns regarding its potential to cause dentine discolouration [193] and produce cytotoxic and inflammatory reactions from the host tissues [194]. Other antimicrobial metallic nanoparticles that have been investigated as root canal irrigants include zinc oxide, magnesium oxide, titanium dioxide, and iron oxide [195, 196].

Chitosan is an organic polysaccharide that is derived from chitin, a natural component found in shrimp and crab shells [197]. Chitosan nanoparticles exhibit excellent biocompatibility as well as promising antibiofilm and antimicrobial properties [198, 199]. Chitosan nanoparticles were found to potently kill planktonic and biofilm *E. faecalis* cells [200]. Studies have demonstrated its capacity to remove the smear layer [199, 201] and stabilize dentine by providing inhibiting collagenase degradation [202]. Chitosan nanoparticles have also been functionalized with photosensitizers such as Rose Bengal and methylene blue to enhance antibiofilm efficacy *via* photodynamic activation [203, 204].

Intracanal medicaments with calcium hydroxide nanoparticles have been developed to help improve its diffusivity and antibacterial activity [205]. Studies have reported that nano-calcium hydroxide was able to penetrate deeper into dentinal tubules and was more effective against *E. faecalis* compared to conventional calcium hydroxide [205–207]. These novel intracanal medicaments were also found to have less detrimental on the fracture resistance of the root dentine [205]. Nanosized calcium silicate compounds have also been investigated as novel intracanal disinfectants by acting as drug delivery vehicles allowing the sustained release of antimicrobial compounds [208, 209]. Mesoporous calcium silicate nanoparticles combined with silver nanoparticles were able to inhibit the growth of both planktonic and biofilm forms of *E. faecalis* [209, 210]. Studies have also suggested their applicability as novel root canal sealers due to their bioactive and osteogenic properties [211–214]. Other nanoparticles with sustained antibiofilm activity that have been explored as root canal sealers include quaternary ammonium polyethyleneimine [215] and quaternary ammonium methacrylate nanoparticles [216].

#### Light Activated Disinfection

In general, disinfection strategies that utilize laser or light can be divided into three groups: direct laser irradiation, photodynamic therapy, and laser activated irrigation [217]. Research into traditional forms of laser endodontics, such as direct laser irradiation, have fallen out of favor because of detrimental thermal effects on the root dentine [218] and a lack of *in vivo* microbiological evidence to support its use as a root canal disinfectant strategy [219]. Furthermore, irrigation with 1% sodium hypochlorite had a more remarkable antibacterial effect in root canals infected with *E. faecalis* when compared to direct laser irradiation [220]. Instead, the focus has shifted toward laser activated irrigation and photodynamic therapies, both of which appear to be of promising value as disinfection adjuncts.

Laser activated irrigation harnesses the ability of certain wavelengths to react with water molecules to instigate the cavitation effect. This produces bubble expansion followed by implosions and shock waves, resulting in turbulent flow that helps distribute the irrigants into anatomical complexities [221]. An *in vitro* study reported that Erbium:YAG (Er:YAG) laser activated irrigation was better at eliminating *E. faecalis* biofilms than ultrasonic activated or syringe irrigation [222]. A variation of laser activated strategies is photon-induced photoacoustic streaming (PIPS). Whilst it also operates using the principles of hydrodynamic cavitation, the main difference is that the laser tip is specially designed to allow it to be positioned the pulp chamber without needing to advance into the root canal system [223]. Several studies have shown PIPS was effective at removing biofilm and the smear layer [224, 225]. Dentinal tubule disinfection was more favorable using PIPS compared to ultrasonic irrigation and Er:YAG laser activation, however none were unable completely eliminate bacterial biofilm and tissue remnants [226].

Photodynamic therapy involves the use of light at a specific wavelength that interacts with a photo-absorbing photosensitizer to excite its electronic layers to a so-called triplet state. In this state, the photosensitizer may react with molecular oxygen and generate reactive oxygen species that elicit an antibacterial effect by degrading microbial lipids, proteins, and DNA [227, 228]. Several options exist for photosensitizers such as methylene blue [204], Rose bengal-conjugated chitosan [229], toluidine blue [230], and indocyanine green [231]. Studies have highlighted the antibiofilm potential of photodynamic therapy for multiple species biofilms [232, 233]. Photoactivated rose bengal-conjugated chitosan was also able to inactivate endotoxins *in vivo* [229]. Notably, Curcumin, the compound responsible for the yellow hue of turmeric or *Curcuma longa*, was found to effectively eliminate biofilm bacteria when applied as a photosensitizer [234]. A systematic review concluded that although there was a paucity of clinical studies, the available evidence suggests photodynamic therapy is able to effectively reduce the microbial load and can be a worthy adjunct to current disinfection protocols [235].

#### Natural Compounds (Phytotherapy)

Natural therapeutic options have sparked the interest of researchers in the search of novel root canal disinfectants. A variety of herbal and plant derived compounds have been identified as potential root canal disinfectants owing to a range of favorable attributes including antibacterial, antifungal, and biocompatible properties. Some of these compounds are found in commonly encountered herbs and spices. *Cinnamomum zeylanicum, Syzygium aromaticum*, and *Ocimum sanctum*, also known as cinnamon, clove and holy basil, each showed antimicrobial activity against *E. faecalis* in both planktonic and biofilm forms [236]. The essential oil constituents, particularly eugenol, may be responsible for some of the antimicrobial properties of these herbal compounds [236]. Specifically, *trans*-cinnamaldehyde, a phenylpropanoid compound, commonly found in cinnamon, was recently reported to eradicate biofilm cells as potently as sodium hypochlorite and more importantly prevented repopulation of bacteria [114], in contrast to chlorhexidine which could not prevent biofilm recovery [138]. This is a significant finding as this is the first evidence that a natural molecule can kill biofilm cells and prevent reinfection with the same potency as sodium hypochlorite in clinically relevant time frames.

Other plant extracts whose applications traditionally have roots in from Ayurvedic, Chinese or other historical forms medicines include berberine, *Morinda citrifolia* and Triphala. Berberine, a quaternary ammonium salt found in a variety of plants, was reported to be more efficacious against a multi-species biofilm model when compared to 5.25% sodium hypochlorite and 2% chlorhexidine [237]. Triphala is a mixture of the fruits from three plant species *Emblica officinalis, Terminalia bellirica*, and *Terminalia chebula*. It was proposed as an herbal alternative to conventional irrigants owing to its ability to completely inhibit *E. faecalis* biofilm formation [150]. The juice from the *Morinda citrifolia*, colloquially known as noni juice, exhibited greater antimicrobial efficacy against *E. faecalis* in dentinal tubules than calcium hydroxide. However, 2% chlorhexidine reigned superior in regard to the antimicrobial effect [238].

Propolis is the resinous product of bees derived from sap and other botanical material. Kandaswamy et al. [238] also reported that propolis had comparable inhibitory effects against *E. faecalis* when compared to *Morinda citrifolia*. When applied as an intracanal medicament, it was found to have better antimicrobial activity against *E. faecalis* compared to calcium hydroxide [239]. Propolis-loaded nanoparticles exhibited low cytotoxicity and antimicrobial efficacy against *E. faecalis, Streptococcus mutans* and *Candida albicans* [240].

#### Antimicrobial Peptides

Antimicrobial peptides have recently gained attention for their potential therapeutic applications in disinfection. This diverse group of biomolecules are involved in various host-defense mechanisms and are also responsible for immunomodulation [241]. In general, these peptides disrupt and destabilize the bacterial membrane integrity and cellular functions, eventually leading to cell death. This enables their broad-spectrum antimicrobial activity including drug-resistant strains [241, 242]. A myriad of antimicrobial peptides have been reported to possess promising antibiofilm and antimicrobial efficacy against various microorganisms implicated in root canal infections. One of the first antimicrobial peptides to be discovered and subsequently gain attention for its potential biomedical applications is nisin [243]. Nisin used as an irrigant was found to reduce *E. faecalis* growth and disrupt its biofilm structure [244]. Peptide GH12 exhibited multimode mechanisms against *E. faecalis* by influencing its genetic expression and virulence whilst also proving to be bactericidal *in vitro* and *ex vivo* [245]. Peptide DJK-5, with or without chlorhexidine was shown to have efficacy against multispecies biofilms [246]. When mixed with EDTA, peptide DJK-5 likewise exhibited superior antibacterial properties against *E. faecalis* biofilms compared to another peptide 1,018 [247], which when combined with chlorhexidine, exhibited a less pronounced antibacterial effect and required longer periods of exposure [246]. The latter peptide nonetheless has the potential to modulate the host immune response and inflammation, which may provide another dimension of benefit [248].

## Conclusions and Call for Future Research

This review focused on the microbial aspect of root canal infections and disinfection strategies, with focus on intra-radicular microbiological challenges. Omics-approaches have considerably enhanced our appreciation of the taxonomic diversity within root canal communities, and to a lesser extent, of the functional and metabolic pathways of bacterial survival within root canals. These aspects can be further addressed by metatranscriptomic, metaproteomic, and metabolomic approaches [93] that may well reveal altered functional profiles in disease-associated communities that underpin pathogenicity.

Such high-throughput approaches would additionally benefit from standardization efforts, in sampling and controls' design for instance. This would facilitate inter-studies comparisons and help dodging methodological pitfalls [249]. Also, further investigations may gain relevance by attaching clinical issues to the community-based investigations, rather than remaining solely descriptive of microbial communities. As examples, could we exploit microbiome data from deep caries or exposed pulps in order to guide treatment strategy toward vital pulp approaches or pulpectomy? Could the root canal microbiota have a prognostic value on periradicular healing? Why is it that pulpal inflammation and periradicular disease may sometimes develop unnoticed by the patient, and in other instances assume excruciating clinical presentations that may lead to life-threatening infectious complications? Whereas the state of the local immune system was shown to contribute to curbing the process [250, 251], little is known of the microbiological factors responsible for acute infections.

As mounting evidence supports associations between endodontic infections and systemic conditions such as diabetes mellitus or cardiovascular diseases [8–10], could we identify microbial signatures able to predict such systemic outcomes? Lastly, in order to combat the biofilm-mediated etiopathogenesis of root canal infections, future research may be directed toward novel anti-biofilm strategies alongside targeting the microbial impetus, such as quorum sensing inhibition, EPS dispersion, biofilm disassembly, and inhibition of signaling systems and macromolecule synthesis, which may pave the way to a variety of novel endodontic disinfection therapies [252]. This may pave the way for the development of more holistic, and potentially more powerful, endodontic disinfection therapies. Addressing these still-open questions may contribute to provide a framework for improving diagnostic procedures, therapeutic regimens and reduce the incidence of adverse clinical evolutions.

## Author Contributions

PNe and GB: conception, design, manuscript—editing, and final draft. JW, DM, and PNä: literature review and manuscript preparation—original draft. All authors contributed to the article and approved the submitted version.

## Conflict of Interest

The authors declare that the research was conducted in the absence of any commercial or financial relationships that could be construed as a potential conflict of interest.
